# Successful bail-out of a massive air embolism during catheter ablation for atrial fibrillation

**DOI:** 10.1016/j.hrcr.2022.12.006

**Published:** 2022-12-10

**Authors:** Satoko Shiomi, Michifumi Tokuda, Seigo Yamashita, Takayuki Ogawa, Teiichi Yamane, Michihiro Yoshimura

**Affiliations:** Department of Cardiology, The Jikei University School of Medicine, Tokyo, Japan

**Keywords:** Atrial fibrillation, Catheter ablation, Complication, Air embolism, Aspiration

**Key Teaching Points**

• Aspiration is effective for a large amount of air intrusion during catheter ablation of atrial fibrillation.
• To prevent embolization of the arterial system in case of rapid air intrusion, it is safer to change the sheath in the right atrium.
• In case of air intrusion, maintaining the patient in the supine position prevents air dispersion to the brain.

## Introduction

Catheter ablation is an effective treatment for atrial fibrillation (AF). With the evolution of catheters, the incidence of periprocedural complications has decreased. Air embolisms are a severe and sometimes life-threatening complication.^1^ We experienced a case involving the successful bail-out—without any residual neurological disability—of a massive air embolism filling the ascending aorta and right coronary artery during radiofrequency catheter ablation of AF.

## Case report

A man in his 60s with hypertension developed dyspnea on exercise and was diagnosed with persistent AF. He was started on edoxaban (60 mg, once daily). His body mass index was 24.5 kg/m^2^. No sleep apnea syndrome had been pointed out. A family doctor referred him to our hospital for catheter ablation of AF.

**Figure 1:**
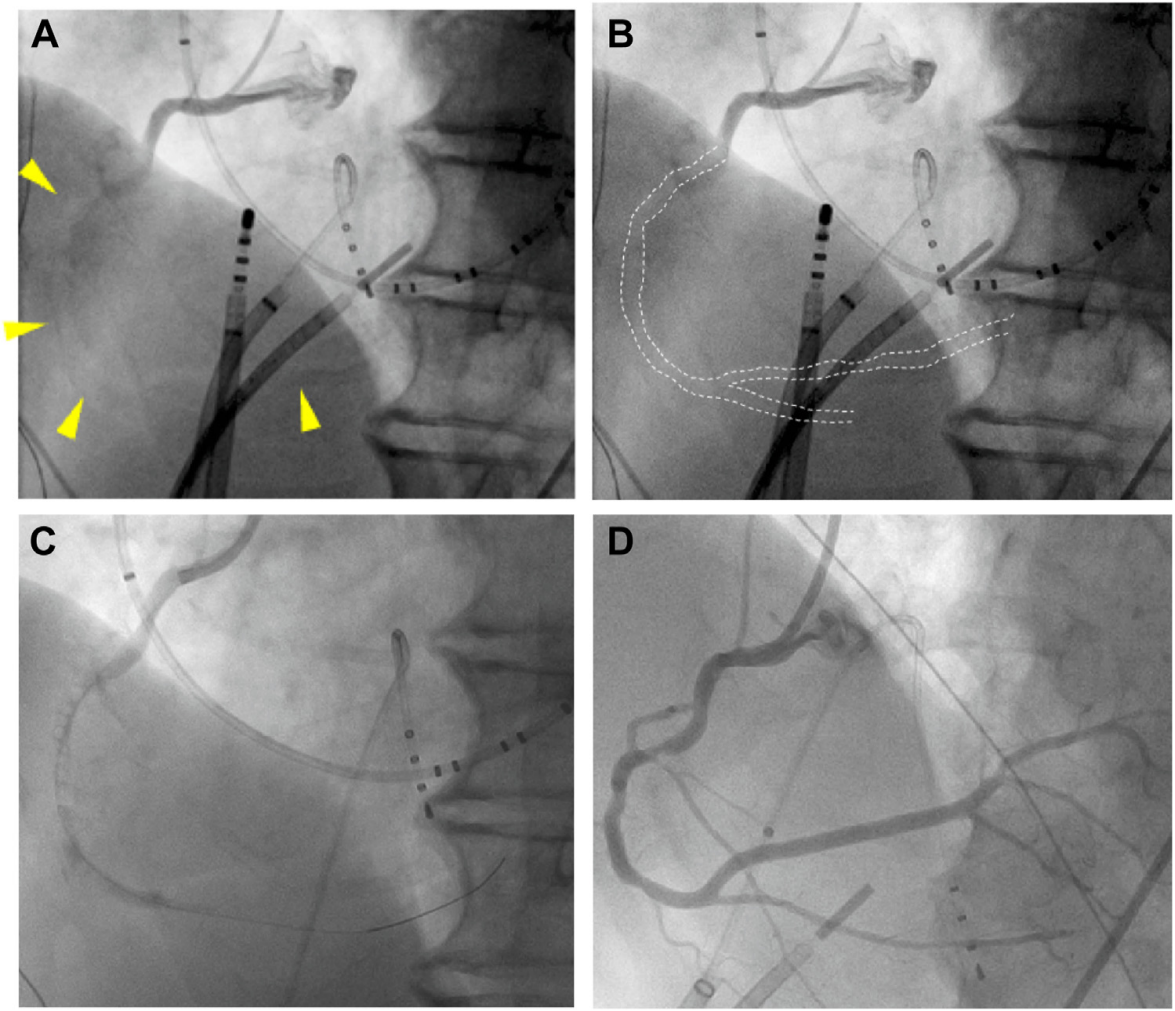
Coronary angiography. **A, B:** The right coronary artery was entirely filled with a large amount of air. **C:** Air in the right coronary artery was aspirated, and partial coronary blood flow was improved. **D:** Air embolism of the right artery completely recovered after suction.

One day before the ablation procedure, transesophageal echocardiography confirmed the absence of left atrial appendage thrombus. On the day of the ablation procedure, the patient presented in AF with an average heart rate of 58 beats/min. The procedure was performed under deep sedation using propofol and flunitrazepam with the assistance of adaptive servo-ventilation (ASV). The procedure was consistently performed in the supine position. After obtaining right femoral venous access (9F and 2 8F long SL0 sheaths [Abbott, St. Paul, MN] in the right femoral vein), heparin was administered to maintain an activated clotting time of 300–350 seconds. A single transseptal puncture was performed using intracardiac echocardiographic and fluoroscopic guidance. AF was restored to sinus rhythm after defibrillation with 150 J. Under sinus rhythm, each pulmonary vein was successfully isolated by a radiofrequency catheter (Tacti Catheter SE D-D; Abbott). The cavotricuspid isthmus line was ablated, and a bidirectional block was confirmed. The SL0 sheath was replaced in the left atrium (LA) by a deflectable sheath (Agilis; Abbott) for LA linear ablation. After that, the patient suddenly became agitated, hypotensive, and bradycardic with ST elevation of II, III, and aVF on a 12-lead electrocardiogram. Intracardiac echography revealed no epicardial effusion; however, left ventricular inferior wall hypokinesis was observed. Subsequently, cardiac arrest occurred, and emergency coronary angiography under pacing (pacing cycle length = 600 ms) from the right ventricular apex was performed. Fluoroscopy showed a translucent right coronary artery (RCA) (Figure 1A and 1B), which indicated that the RCA was entirely embolized by a large amount of air. The patient’s head was held down to avoid raising it above the horizontal level. An aspiration catheter (Thrombuster 7Fr GR; Kaneka Medical, Tokyo, Japan) was used to aspirate a large amount of air; the coronary angiography revealed residual air in the RCA (Figure 1C), and a smaller aspiration catheter (Thrombuster 6Fr GR; Kaneka Medical) was used to attempt aspiration from the distal RCA and to inject nitroprusside hydrate. The air in the RCA was removed entirely by further washout with saline, and the ST elevation improved (Figure 1D). At this point, air retention was suspected in the anterior portion of the ascending aorta (between the aortic root and arch, which is the highest level in the supine position). A 5F pig-tail catheter and a 5F Judkins Right (JR4) catheter were inserted from the femoral artery to aspirate air in the ascending aorta. Then, the air in the ascending aorta appeared to be entirely removed. Further ablation procedures ceased. The patient was admitted to the coronary care unit ward.

After the procedure, his consciousness rapidly recovered. A certified neurologist performed a systematic clinical neurological examination and noted no abnormal neurological findings. Diffusion-weighted magnetic resonance imaging showed a small high-intensity area (2.0 × 2.2 mm) in the right corona radiata that was suspicious for acute cerebral infarction. No residual air was observed on cerebral, chest, or abdominal computed tomography. The peak creatine kinase (CK) and CK-MB levels were 355 U/L (normal levels 59–248 U/L) and 32 U/L (normal levels 0–5 U/L), respectively, and he was discharged without a residual disability on the fourth postoperative day. The patient had a recurrence of AF 1 year later, underwent repeat ablation, and remained AF-free.

## Discussion

### Mechanisms of air contamination

Massive air embolism during AF ablation is rare. There have been very few reports of the treatment of a massive air embolism in the RCA and the ascending aorta without any residual neurological disability during catheter ablation of AF.^2^ Several mechanisms are known to increase the risk of air intrusion via the sheath: (1) repeated catheter changes, (2) quick removal of the catheter, (3) apnea, and (4) negative pressure of intrathoracic pressure. Repeated catheter changes and fast flushing of the long sheath can push air into the cardiovascular system.^3^ Rapid catheter removal from the sheath creates a vacuum and sucks air through the hemostatic valve. This risk increases when the sheath and catheter sizes differ significantly.^4^ Particular care should be taken as to whether the tip of the sheath is in contact with a wall. AF ablation is usually performed under general anesthesia or deep sedation. When the procedure is performed under deep sedation, apnea also affects air intrusion via the sheath. When the intrathoracic pressure becomes negative, air can be sucked through the hemostatic valve. The longer the apnea duration, the greater the negative intrapleural pressure becomes. However, the patient was not obese, he had no history of sleep apnea, and his respiratory status was stable intraoperatively under ASV. ASV sends air during apnea to stabilize breathing, eliminating the sudden negative pressure after apnea. In this patient, sheath exchange in the LA can cause air embolization into the arterial system. Performing the sheath exchange in the right atrium and rethreading the atrial septum is safer. It is possible that the catheter was quickly removed from the sheath. The sheath valve may have been incompletely closed when the catheter was removed, and when this coincided with deep inspiration, a large amount of air may have been momentarily sucked into the LA.

### Management of air embolism

**Figure 2:**
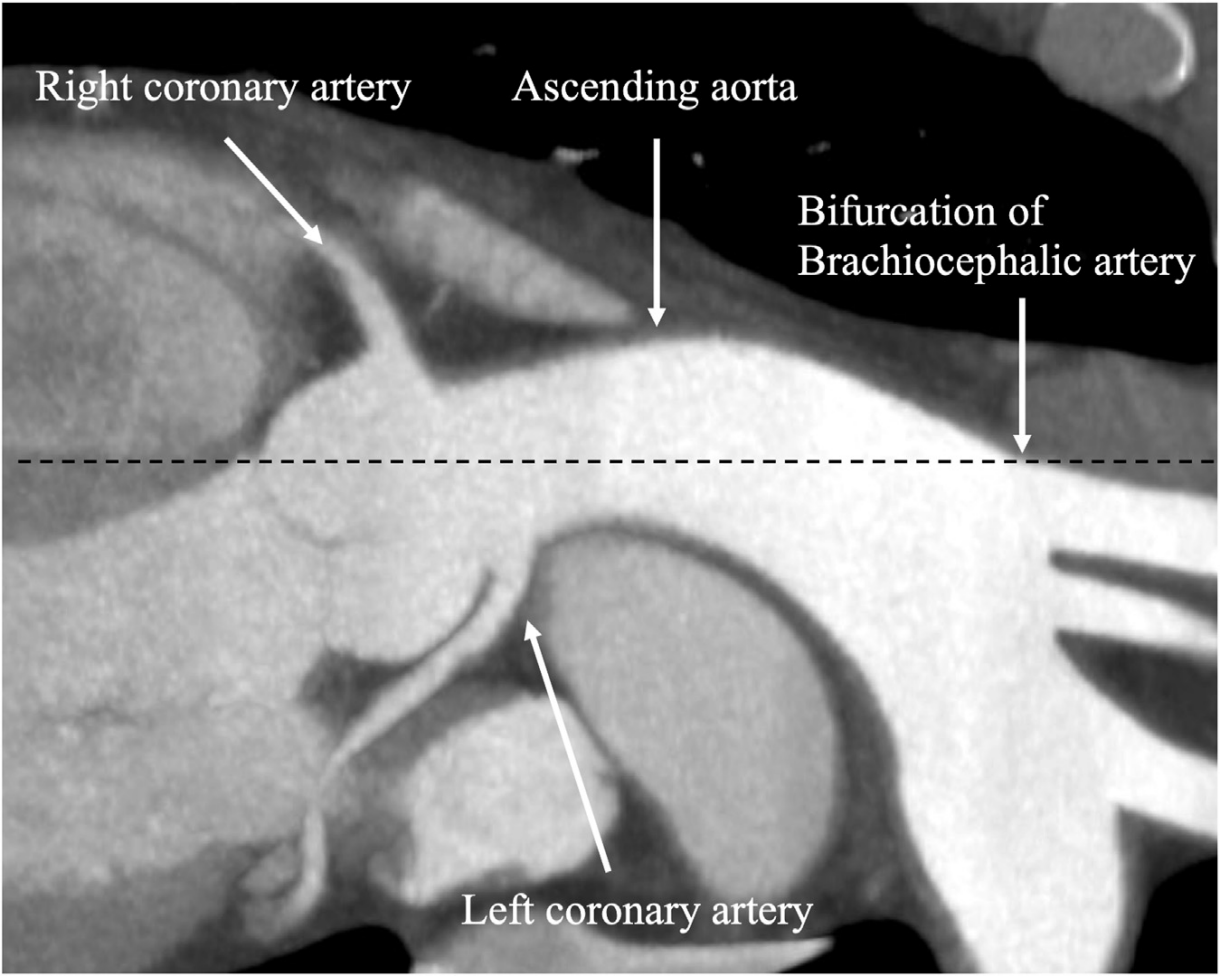
Positional relationship between aortic vessels and branches. Contrast computed tomography shows that the anterior wall of the ascending aorta was located higher than the brachiocephalic bifurcation in the supine position. The dotted line represents the height of the bifurcation of the brachiocephalic artery.

If the volume of intruded air is small, air embolism is usually improved only with supportive care, such as pacing and the administration of vasodilators. However, if the volume of intruded air is significant, catheterized aspiration is recommended. An increase in mean arterial pressure pushes it into the coronary microcirculation, and dilation of the coronary arteries improves the no-reflow phenomenon. If circulatory collapse occurs, hemodynamic support, such as an intra-aortic balloon pump, is required. We have shown that massive air embolisms in the coronary arteries can be entirely removed with the aspiration catheter. Direct air aspiration from the RCA and crushing large occlusive air by the forceful injection of saline into the RCA have been reported.^5^ Air in the ascending aorta can also be removed. Since air is usually stored in the anterior portion of the ascending aorta, a femoral artery approach allows the catheter better access to the site of air storage. Although there are few reports of air being recovered with pig-tail catheters, we believe that pig-tail catheters have many small holes and are effective for aspirating air that was widely stored in the aorta and the LA. When air intrusions are identified, it is essential to avoid cerebral air embolism. Maintaining the patient in the supine or Trendelenburg position is paramount because the anterior wall of the ascending aorta is anatomically located higher than the carotid bifurcation in the supine position (Figure 2). Thus, air accumulates in the anterior aspect of the ascending aorta. If the head is raised, this air will be dispersed into the brain.

Future measures for preventing air intrusion include the following: (1) check for sheath malfunctions at the start of the procedure; (2) obliterate air from inside the sheath to prevent air infiltration; (3) perform sheath exchange in the right atrium; (4) remove the catheter slowly, and (5) avoid deep sedation that causes apnea and avoid excessive intravascular negative pressure.^6^

## Conclusion

We experienced a case of massive air embolism of the RCA and ascending aorta during RF catheter ablation for persistent AF in which emergency catheter treatment rescued the patient without any residual disability.
